# Central airway obstruction: is it time to move forward?

**DOI:** 10.1186/s12890-022-01862-x

**Published:** 2022-02-19

**Authors:** Fernando Guedes, Mariana V. Branquinho, Ana C. Sousa, Rui D. Alvites, António Bugalho, Ana Colette Maurício

**Affiliations:** 1 Centro de Estudos de Ciência Animal (CECA), Instituto de Ciências, Tecnologias e Agroambiente (ICETA) da Universidade do Porto, Praça Gomes Teixeira, Apartado 55142, 4051-401 Porto, Portugal; 2 Departamento de Clínicas Veterinárias, Instituto de Ciências Biomédicas Abel Salazar (ICBAS), Universidade do Porto (UP), Rua de Jorge Viterbo Ferreira, nº 228, 4050-313 Porto, Portugal; 3 Pulmonology Department, Bronchology Unit, Centre Hospitalier du Luxembourg, Luxembourg, Luxembourg; 4 CUF Tejo Hospital e CUF Descobertas Hospital, Lisbon, Portugal; 5 Centro de Estudos de Doenças Crónicas (CEDOC), NOVA Medical School, Lisbon, Portugal

**Keywords:** Airway stents, Central airway obstruction, Interventional bronchology, Regenerative medicine

## Abstract

**Introduction:**

Central airway obstruction (CAO) represents a pathological condition that can lead to airflow limitation of the trachea, main stem *bronchi*, *bronchus intermedius* or *lobar bronchus*.

**Main body:**

It is a common clinical situation consensually considered under-diagnosed. Management of patients with CAO can be difficult and deciding on the best treatment approach represents a medical challenge. This work intends to review CAO classifications, causes, treatments and its therapeutic limitations, approaching benign and malign presentations. Three illustrative cases are further presented, supporting the clinical problem under review.

**Conclusion:**

Management of CAO still remains a challenge. The available options are not always effective nor free from complications. A new generation of costume-tailored airway stents, associated with stem cell-based therapy, could be an option in specific clinical situations.

## Introduction

Central airway obstruction (CAO) is defined as a pathological condition that leads to airflow limitation of the trachea, main stem *bronchi*, *bronchus intermedius* or *lobar bronchus*. It represents an important clinical situation that affects both adults and children. The exact incidence is, to the authors knowledge, unknown, but is widely accepted by the medical community that this disease is underdiagnosed [1]. While a surgical approach is considered the *gold-standard* treatment for most clinical situations, especially in benign etiologies, multiple factors concerning the underlying disease, the degree and type of CAO, and the patient’s condition may discourage this option. Interventional bronchoscopy emerged more than 120 years ago with Gustav Killian's works in bronchoscopy, and has been validated as a compelling treatment with immediate effects in CAO for the last 40 years [2, 3]. Regarding CAO, the scientific and medical communities currently face several limitations that arise from the anatomical, structural, and physiological characteristics of the trachea and main bronchi. The normal adult trachea is usually a rigid 11-14 cm tube supported anteriorly by C-shaped cartilaginous rings and posteriorly by smooth muscle, and this structure as a whole has some, although restricted, mobility during the respiratory process [4]. In older patients or those with hyperinflation, the posterior membranous wall may collapse during expiration. Any variation on the trachea diameter during the normal respiration cycle or any endobronchial changes, such as injury or secretions, may impair the ventilatory dynamic and affect the Reynolds number (transition from laminar to turbulent flow with increase resistance), thus resulting in the need for higher-pressure difference to maintain a normal flow rate [5, 6]. This will be reflected in the development of symptoms, especially in patients with a marked reduction in the cross-sectional area.

CAO is caused by several disorders and the first step for its approach is a clear etiological evaluation (Table 1).

**Table 1:** The main causes of tracheobronchial stenosis according to different etiologies

Acquired	**Tumour**	**Systemic diseases**
	Cystic carcinomas	Granulomatosis with polyangiitis
	Bronchial carcinoid tumours	Amyloidosis
	Primary squamous cell carcinoma of the trachea	Sarcoidosis
	Adenocarcinoma of the trachea	Systemic erythematous lupus
	Primary lung cancer	Relapsing polychondritis
	Metastasis	Tracheobronchopatia osteochondroplastica
	**Trauma**	**Tracheobronchomalacia**
	Previous instrumentalization	
	Post-intubation	Extrinsic compression
	Post-tracheostomy	Abnormalities of the aortic arch
	Foreign body	Thymic neoplasm
	Thermal	Lymphoma
	Caustic	Mediastinal or hilar increased lymph nodes
	Radiation	Advanced esophageal cancer
	Anastomotic	Diffuse goiter
		Postpneumonectomy syndrome
	**Infection**	Hematomas, abscess, empyema
	Tuberculosis	
	Syphilis	**Other causes**
	Typhoid fever	Idiopathic
	Diphtheria	Gastroesophageal reflux
	Fungal infection	
Congenital	Membranous (fibrous tissue, granulation tissue)	
	Cartilaginous (cartilage deformity)	
	Combined	

Congenital or acquired CAO may result from intrinsic stenosis or extrinsic compression, leading to fixed obstruction, or caused by cartilage or *pars membranous* flaccidity, with dynamic obstruction. In the early twentieth century, infection and trauma were the main causes of CAO. In the 60 s, with the advent of intensive care units and the common practice of prolonged orotracheal intubations, post-intubation tracheal stenosis (PITS) and post-tracheotomy (PSST) tracheal stenosis incidence increased. Malignant causes of CAO, which have been reported in up to 20–30% of lung cancers in older studies [7], do not reflect contemporary lung cancer epidemiology. These rates are now lower, with the progressive change in lung cancer incidence and prevalence, favouring peripheral adenocarcinoma development [8]. A recent retrospect analysis of 342 baseline CT scans of lung cancer revealed 13% of patients having CAO [9].

**Fig. 1:**
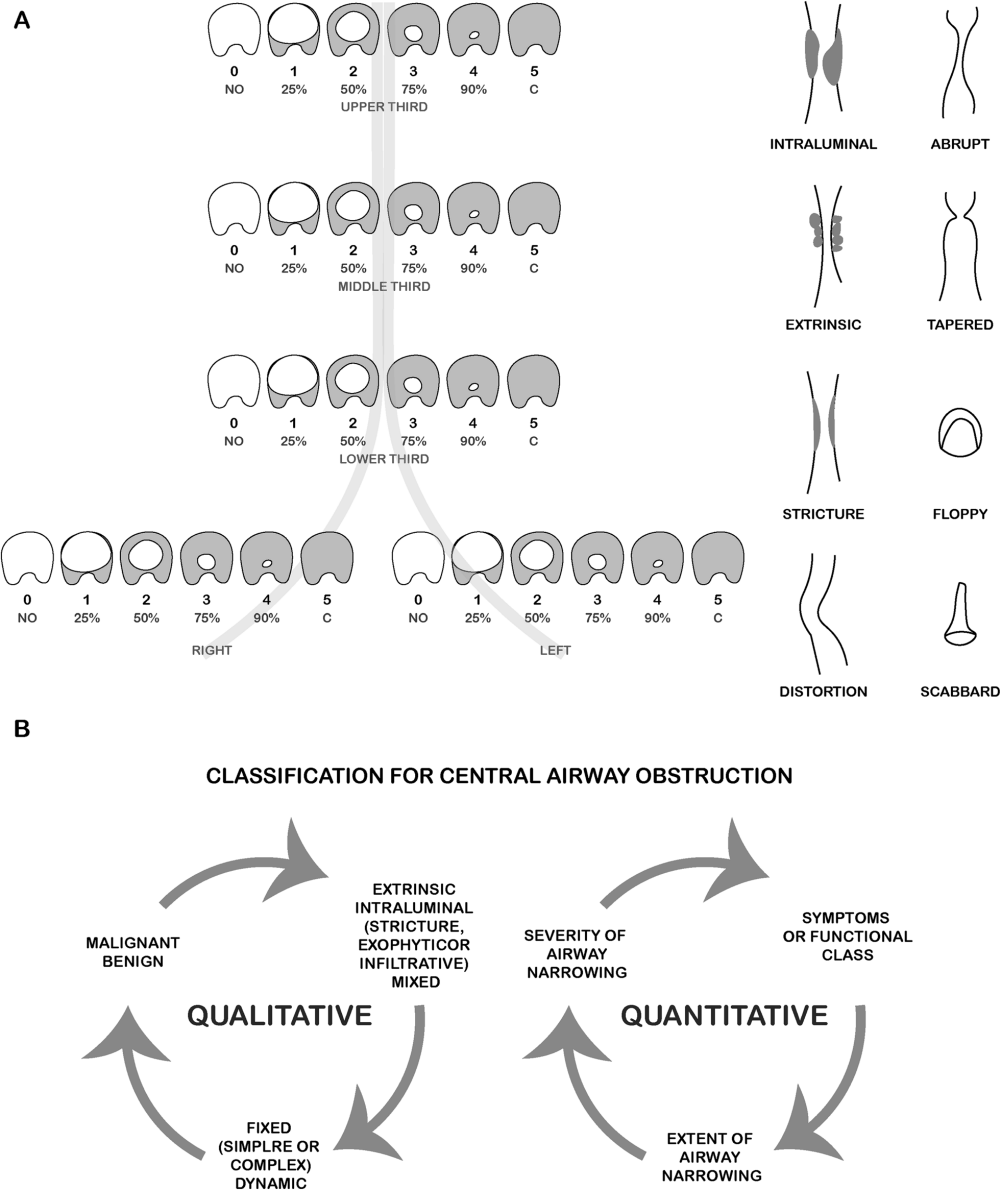
**A** Classification according to type, grade and site of stenosis proposed from Freitag et al., adapted from [15]. **B** Qualitative and quantitative classification proposed by Murgu and Colt, adapted from [16]

Since CAO has multiple etiologies and different expressions, a clear definition and classification is of utmost relevance, as to estimate prognosis and determine the best therapeutic options [7]. A classification system based on histologic findings, pathological mechanisms, dynamics, functional impairment severity of airway narrowing and extend of airway abnormalities is crucial. The multidisciplinary involvement has led to the development of several systems with distinct criteria. Regarding laringotracheal stenosis, they were first classified by Cotton in 1984 based on the cross-sectional area of the stenosis [10]. In 1987, Grundfast et al. using the same criteria, upgraded the system looking for the predictive outcome [11]. In 1992 McCaffrey proposed a classification based on the vertical extend of the stenosis and predicting decannulation centred on anatomic location and extend [12]. Grillo et al. in 1993 reviewed the anatomic location and the possibility of anastomosis. In 1994, the Myer-Cotton Airway Grading System for subglottic stenosis was created and became routinely used to predict interventional success [13]. The four degrees of this system consider an obstruction of 0–50% for grade I, 51–70% for grade II, 71–99% for grade III, and complete luminal obstruction for grade IV [14]. The proposal of Lano et al. in 1998 was based on subsites involved. All those studies were focused on surgical approaches and outcomes based mainly on treatable anatomic premises, excluding extensions beyond the trachea and the possibility of endobronchial treatment. In 2007, Freitag et al. published a classification system dividing stenosis into structural and dynamic types, in addition to categorizing its degree and location (Fig. 1A). This classification was designed for grading tracheal stenosis from a pulmonologist’s perspective, but the degree of severity was not justified clinically. On the other hand, a histological classification was not included [15].

In 2010, Murgu and Colt made a different proposal, where a qualitative and quantitative classification was considered [16]. The gaps observed in Freitag et al. classification were also filled (Fig. 1B). The idea of creating an ideal and reproductive classification for CAO has not yet been fully completed and consensus has not yet been reached. In the future, ongoing studies will standardize nomenclatures and find descriptors that include the degree of stenosis and patient´s functional status, histologic and morphologic types, extension, localization, and mechanism of obstruction.

The European Laryngological Society (ELS) published a consensus paper in 2015 that offered a new classification system of benign laryngo-tracheal stenosis (LTS) in adults and children [17]. It included a routine five-step endoscopic evaluation of patients, to assess the grade of stenosis (Cotton– Myer grade I–IV), number of subsites involved (“a–d” for one to four of supraglottic, glottic, subglottic, and tracheal segments involved), and an additional plus sign (“+”) for patients with severe airway or medical comorbidities. The focus of this classification was to access the surgical outcome of patients with LTS.

## Etiology

### Congenital CAO

In the adult population, congenital disease itself as a cause of CAO is a very rare condition [18]. When identified, it may result from posterior fusion of the tracheal rings. In children, it is frequently associated with congenital cardiovascular abnormalities, the most common being the compression of anterior tracheal wall for an anomalous bifurcation of the innominate artery [19].

### Acquired CAO

#### Tracheobronchial trauma/iatrogenic

Acquired non-malignant CAO is most frequently caused by iatrogenic tracheal trauma after prolonged intubation or tracheostomy tube placement. It is estimated that the incidence of PITS ranges from 6 to 21% and PTS 0.6 to 21% [20, 21]. PITS occurs mostly in the anatomical area of the inflated cuff and is related to pressure induced ischemic injury of the mucosa and cartilage. On the other hand, the inflammation caused by a foreign body may result in granulation tissue followed by fibrosis. Web-ring stenosis is the most common form [22]. With low-pressure cuffs and maintaining a pressure under 30 mmHg, it was possible to decrease the incidence of post-intubation stenosis to 1% and of post-tracheotomy to 10–15% [23]. The duration of intubation also impacts the frequency and severity. PSST may be above the stoma, at the level of the stoma, at cuff site or at the tip of the cannula. It results from a process of tissue repairing and formation of granulation tissue around the stoma. The most common type is granulation tissue formation at the stoma site. Stenosis due to cartilage fracture, tracheomalacia and web-like can also be seen [24]. Factors associated with PITS and PSST are the level of stoma, previous tracheal procedure, low blood pressure, severe respiratory failure, sepsis, concomitant autoimmune disease, sleep apnea syndrome,obesity, diabetis,high-dose corticosteroid therapy previous radiation and intubation > 48 h [25]. Post-transplantation bronchial stenosis occurs in 5–30% of patients and results from all repair process of anastomosis [26]. These anastomosis are vulnerable for several reasons: rejection may occur, and immunosuppression often results in infection and/or ischemia, especially in the first 4 weeks, which is the necessary time for bronchial arteries to re-establish full perfusion. Other causes of tracheobronchial stenosis include lobectomy, and sleeve and tracheal resections [27].

Trauma may also arise from thermal and chemical injuries or treatment with radiotherapy, causing localized stenosis.

Foreign body aspiration is more common in children and elderly and can lead to acute CAO. If the foreign body is not large enough to significantly occlude the lumen, granulation may emerge and cause obstruction. If the foreign body has an organic nature, a chemical bronchitis can occur [28].

#### Infection

Tuberculosis is the most common cause of post-infection stenosis in adults, mainly in high-prevalence geographic regions. It is usually caused by endobronchial involvement or lymph node fistulation trough adjacent bronchi. Early diagnosis and treatment with anti-tuberculosis medications typically prevents this situation [29]. Laryngotracheobronchitis (croup) is an acute viral respiratory disease which occurs usually in children under 6 years old and is rare in adults. Acute bacterial tracheitis due to *S. Aureus* sometimes can occur as over-infection after a viral episode and may cause CAO [30]. Laryngeal scleroma is a chronic and progressive granulomatous infection caused by *Klebsiella rhinoscleromatis,* a gram-negative bacteria endemic in Africa, Asia and South America, and may cause airway obstruction [31]. Other conditions like diphtheria, syphilis and fungal infections, although rare, are recognized causes of CAO [32].

#### Inflammatory and auto-immune diseases

*Granulomatosis with polyangiitis* is a systemic disease characterized by necrotizing vasculitis and granulomas. The airway is involved in 15–55% of cases and can be the only manifestation in 25% of patients [33, 34]. Granulomatosis with polyangiitis may lead to subglottic, tracheal, or bronchial stenosis, and nasal sinus is frequently involved. Laboratory findings may include positive anti-neutrophil cytoplasmic autoantibodies (c-ANCA) but usually there is no link between inflammatory activity and positive c-ANCA. The mucosa’s biopsy is the gold standard method for diagnosis and usually reveals granulomatous inflammation and vasculitis in early disease, while fibrosis ensues later. Unfortunately it is positive in only 5–15% of cases [35]. Systemic immunosuppressive therapy and steroids are the mainstay treatment, but endoscopic management may be required.

*Tracheobronchial amyloidosis* results from the pathological deposition of amyloid in the proximity of the tracheal, bronchial gland acini and blood vessel walls. It may be present as an isolated manifestation or as part of a systemic disease. Tracheal involvement can range from diffuse lesions to masses. Pulmonary manifestations as persistent pleural effusions and parenchymal nodules can be present. Diffuse wall thickening, irregular narrowing of the lumen and calcifications may be seen during bronchoscopy. Diagnosis is determined by biopsy of the lesions showing positive red Congo staining with submucosal extracellular deposits of amyloid protein. There is no effective treatment and radiation therapy in addiction to interventional bronchoscopy techniques and surgery should be considered [36].

*Sarcoidosis* is a multisystem disorder characterized by noncaseating granulomatous inflammation. Although any organ can be involved, the disease most commonly affects the lungs and intrathoracic lymph nodes. The airway can be affected even in the absence of parenchymal involvement. Bronchial wall thickening caused by granulomas and interstitial fibrous tissue may result in smooth or irregular luminal narrowing. The compression due to mediastinal lymphadenopathy is rare. Bronchoscopic findings include cobblestone appearance of the mucosa originated by granuloma, ranging from single to multiple stenosis sites [37]. The diagnosis of sarcoidosis is established based on a compatible clinical picture, evidence of noncaseating granulomas on biopsy, and exclusion of other granulomatous disorders. Treatment with immunosuppressive therapy may be necessary in severe cases. Bronchoscopic procedures are required in some situations [38].

*Relapsing polychondritis* (RP) is a multisystemic autoimmune disease characterized by recurrent inflammatory episodes affecting cartilaginous structures. These structures include ears, nose, peripheral joints, larynx, and tracheobronchial tree. It is more likely between 40 and 50 years and is often associated with another pathologic conditions as systemic vasculitis or connective tissue disorders [30]. The evolution of the disease alternates between an active inflammatory phase and non-acute phase. During disease course, about half patients will exhibit airway involvement including subglottic stenosis, tracheobronchomalacia and tracheobronchial stenosis. Thorax computer tomography (CT) and bronchoscopy reveal major airway collapse, thickening of the trachea and proximal bronchi, thickened calcified cartilaginous rings and tracheal nodularity. The posterior tracheal membrane is normally spared [35]. Usually, specific clinical criteria and bronchoscopic evaluation of airway are enough to diagnose of RP. If a biopsy is performed, inflammatory changes in the cartilage are normally present. Medical management of RP consist in control the inflammatory process with steroids combined with methotexate, azatriopine or cyclophosphamide. Some studies propose the use of immunomodularity therapies as etanercept, infliximab or rituximab [39, 40]. Interventional bronchoscopy procedures such as dilating, and stenting may help in specific cases [41].

*Tracheobronchopatia osteochondroplastica* (TO) is a rare benign disease with unknown origin that affects the cartilage rings of the trachea and, in lesser extent, the main bronchi. It occurs over the age of 50 years, and it is characterized by cartilaginous or osseous nodules projecting into the airway lumen, causing serious deformity. In 10% of the cases, the tracheal lumen can be reduced up to 50%. CT scans and bronchoscopy show densely calcified cartilage nodules. If the appearance is typical, no biopsy is needed. TO is a slow benign disease and rarely complicates. In cases of more serious degrees of stenosis, endoscopic laser ablation or stent application may be required [41]

#### Tracheobronchomalacia

Tracheobronchomalacia (TBM) is a condition characterized by diffuse or localized loss of rigidity of the trachea and mainstem *bronchi*, leading to airway collapse. If the affected portion is intrathoracic, the airway obstruction will be accentuated during expiration. More rarely, the affected portion is extra-thoracic and is more accentuated during inspiration. Congenital TBM is described more frequently in children and indicates a defect of the tracheal wall. The disorder can persist into adult life and is designated idiopathic giant trachea or Mounier-Khun syndrome. TBM may also develop along with other congenital conditions such as cystic fibrosis, Marfan syndrome, Ehlers-Danlos syndrome or congenital trachea-esophageal fistula [42]. Acquired TBM is associated with a variety of conditions. Prolonged endotracheal intubation and tracheostomy trauma are the most frequent, but head and neck surgery, trauma, radiotherapy or inflammatory conditions must also be considered [43, 44]. The diagnosis is based on dynamic Three-dimensional CT images and direct bronchoscopic visualization to confirm significant narrowing of airway lumen during forced breathing manoeuvres [45]. Applying continuous positive airway pressure has been proposed as a possible treatment [44]. In patients with diffuse TBM, a silicone stent placement should be considered in preparation for a more definitive treatment such as tracheoplasty. Majed et al. purpose a self-expandable metallic stents (SEMS) trial as a bridge to surgery [46]. Other surgical approach consists in restoring the C shape of the cartilages which is lost in TBM attaching longitudinal strips of splinting material along the membranous walls of the intrathoracic trachea [47].

#### Tumours

Both malignant and non-malignant tumours may cause CAO, either from direct extension of a locally advanced disease or from extrinsic compression. Primary tracheobronchial tumours are infrequent and include adenoid cystic carcinomas, bronchial carcinoid tumours, primary squamous cell carcinoma of the trachea and adenocarcinoma of the trachea [48]. Much frequent are metastases from primary lung cancers. Metastasis from breast, renal, thyroid, ovarian, uterine, testicular carcinomas and melanoma may also occur. It is estimated that 20–30% of patients with primary lung cancer will develop CAO; of those, 35% will die in consequence of asphyxia, hemoptysis and post-obstructive pneumonia [49]. Anatomically, malignant CAO is divided into 3 groups: intra-luminal, extrinsic compression and mixed. The purpose of this classification is based on the type of interventional assessment. The interventional bronchoscopy with palliative intent should be considered in these patients which may benefit from measures to relive dyspnea [2, 50]. Recurrent papillomatosis, resulting from infection of the human papilloma virus, produces lesions with polypoid appearance which can affect larynx, trachea, and bronchi. Endoscopic interventions are an important component, as recurrence is common [51].

#### Extrinsic compression

Most frequently extrinsic compression in children arises from abnormalities of the aortic arch and are defined as vascular rings. Double aortic arch and right sided aortic arch with aberrant left subclavian artery are the most common. Compression of the trachea by large aortic or innominate artery aneurysms may occur. Mediastinal masses rarely cause serious airflow limitations. Approximately 40% of mediastinal masses are malignant and 25% are cystic [35]. Thymic neoplasms and lymphoma, both Hodgkin´s and non-Hodgkin´s, are also common, but neurogenic tumours and teratomas must be considered.

An analogous but rare situation can be caused by the tumefaction of mediastinal lymph nodes due to metastatic or infectious diseases. Involvement of the trachea and left side bronchus by advanced esophageal cancer is common and is associated with a poor prognosis. Retrosternal extension of diffuse goiter may cause extrathoracic or intrathoracic airway obstruction [52]. Laryngoceles, saccular cysts and parathyroid cysts may be also implicated in CAO [53]. Airway narrowing due to osteophytes has been reported [54]. Postpneumonectomy syndrome occurs due to a compression of the left main bronchus between the aortic arch and left pulmonary artery, following a right pneumonectomy [55]. Significant CAO can occur from other infectious or malignant diseases like hematomas, abscess formation, empyema, or other expanding lesions. In most cases the treatment should address the underlying cause, but urgent intervention may be needed in case of respiratory failure. Most of the times this includes airway stent deployment to restore and maintain luminal patency [56, 57].

#### Other causes

Idiopathic tracheal stenosis is a rare inflammatory process that develops frequently in the subglottis or in the upper third of the trachea. It occurs mainly in women, suggesting an important role of estrogens[58]. Other authors suggest that it may be associated with gastroesophageal reflux [59].

## Clinical presentation

CAO is much less common compared to other lower airway diseases. However, the symptoms may be identical, which may lead to misdiagnosis. Because these symptoms can be insidious CAO may be obscured for a long time resulting in a delayed diagnosis and worst outcome. Clinical presentation will depend on the location, extension and degree of obstruction, speed of progression and underlying disease. Other conditions, such as the patient healthiness, will have an important role in progression and outcome. The main symptoms are dyspnea and stridor occurring in 54% of patients as initial complain [45]. These manifestations are more prominent during exercise but may progress to dyspnea at rest. The first usually occurs when the lumen is reduced to 8 mm and the second when it reaches less than 5 mm. Cough, wheezing, diminished sputum clearance and recurrent infection will emerge when it reaches this level of severity. Unlike wheezing, stridor occurs predominantly during expiration and is emphasized by hyperventilation. If the obstruction is mainly thoracic, both inspiratory and expiratory stridor will appear. Hoarseness can be heard and could be a signal of laryngeal involvement. Less common is an initial presentation with acute respiratory distress.

## Diagnostic evaluation

When CAO is suspected, clinical investigations to look for the underlying diagnosis include, in the first instance, thorax CT and blood tests (inflammatory markers, auto immune diseases), lung function tests, other imaging exams [X-ray, CT, magnetic resonance imaging (MRI)] and bronchoscopy. Blood tests are frequently normal or with nonspecific changes, but inflammatory markers, white cell blood count or auto-immune screen can identify underlying diseases [35]**.** As the symptoms, physiological and measurable abnormalities in lung function tests frequently do not become evident until the airway lumen is < 8 mm, which corresponds to an obstruction of more than 80%. In these cases, a variable extra-thoracic obstruction shows flattering of the inspiratory loop, intrathoracic obstruction shows flattering of expiratory loop and fixed obstructions reveal flattering in both. On the other hand, if the cause of CAO is a lung tumour, an abnormal lung function test could not be adequately valorised because of the prevalence of lung cancer in chronic obstructive pulmonary disease (COPD) [60], whereas the lung function tests are usually anomalous. Unless the cause of CAO is an extra-thoracic compression caused by a vascular abnormality or tumour, X-ray usually gives few and unspecific information. The thoracic CT with tracheal protocols is the procedure of choice for imaging the upper airway, with a sensitivity of 97% [35]. With standard chest protocols, tracheal disease is easily underestimated. CT allows to identify the precise anatomical location of the lesion, its characteristics and extension, including the visualization of patency of the distal airway. Using an axial view, it is possible to evaluate the degree of stenosis. Extremely thin slices allow 3-D reconstructions, especially useful to provide pre-management volumetric analyses. Dynamic CT is effective for the diagnosis of TBM. MRI has usually limited value although it can be used to access the surrounding mediastinum, mostly the vascular structures [35]. A direct view of the airway is provided by both flexible and rigid bronchoscopy. They allow to directly observe the extension of airway narrowing, the characteristics of the mucosa, and the specific location. Samplings can be collected for microbiological and cytologic analyses. Because of the risk of precipitating acute and complete obstruction, flexible bronchoscopy should be performed judiciously and by trained physicians with appropriate equipment [61].

Although many different causes of CAO and a relatively well-defined protocol approach, its management is not straightforward nor consensual and the long-time outcomes are far from satisfactory in some cases, as shown in Table 2

**Table 2:** Examples of CAO from different etiologies

	Patient 1 (Fig. 2)	Patient 2 (Fig. 3)	Patient 3 (Fig. 4)
Identification	♀ 81 years old	♀ 66 years old	♂ 61 years old
Etiology of CAO	Post orotracheal intubation	Extrinsic compression caused by a thoracic aorta aneurism	Tumoral extrinsic compression
Medical history	Chronical renal failure Pulmonary hypertension LICU hospitalization 2 years before	HIV+ Pulmonary tuberculosis 30 years before	Smoker with 75 pack-year history
Clinical manifestations	Progressive dyspnea	Progressive dyspnea Pleuritic chest pain	Pleuritic pain Dyspnea Hoarseness
Diagnostic evaluation	Expiratory stridor Lung functions tests normal Thoracic CT scan showed mid-tracheal stenosis > 50% Flexible bronchoscopy confirmed diagnosis	Thoracic CT scan showed thoracic aorta aneurism compressing tracheal and main left bronchus Flexible bronchoscopy confirmed diagnosis	Thoracic CT scan showed voluminous adenopatic conglomerate compressing trachea causing a narrowing of > 80% of trachea and LMB Flexible bronchoscopy showed mucosal invasion and confirmed stenosis Bronchial biopsies revealed small cell lung cancer
Discussion and management	No conditions for surgery Dilatation with rigid bronchoscope unsuccessful Introduction of a silicon Dumon Stent	A silicon Dumon stent was placed in LML as a bridge to endovascular correction of the aneurism	Radiation and chemotherapy were pursued with disappointing results Y silicon Dumon stent was placed as a palliative intention
Clinical evolution	Immediate relief of dyspnea and improvement of QOL Complicated after 1 month with stent migration and infection	Difficult extubation after endovascular correction Recurrent mucus plugs with need of repeated bronchoscopies Complicated with voluminous fistula from LMB to mediastinum	Recurrent mucus plugs with need of repeated bronchoscopies A SEMS was placed instead of Dumon stent trying to avoid the repeated mucous obstruction
Outcome	Died with multi-organic shock from pulmonary origin	Died with infectious complications	Died due to progression of the underlying disease

**Fig. 2:**
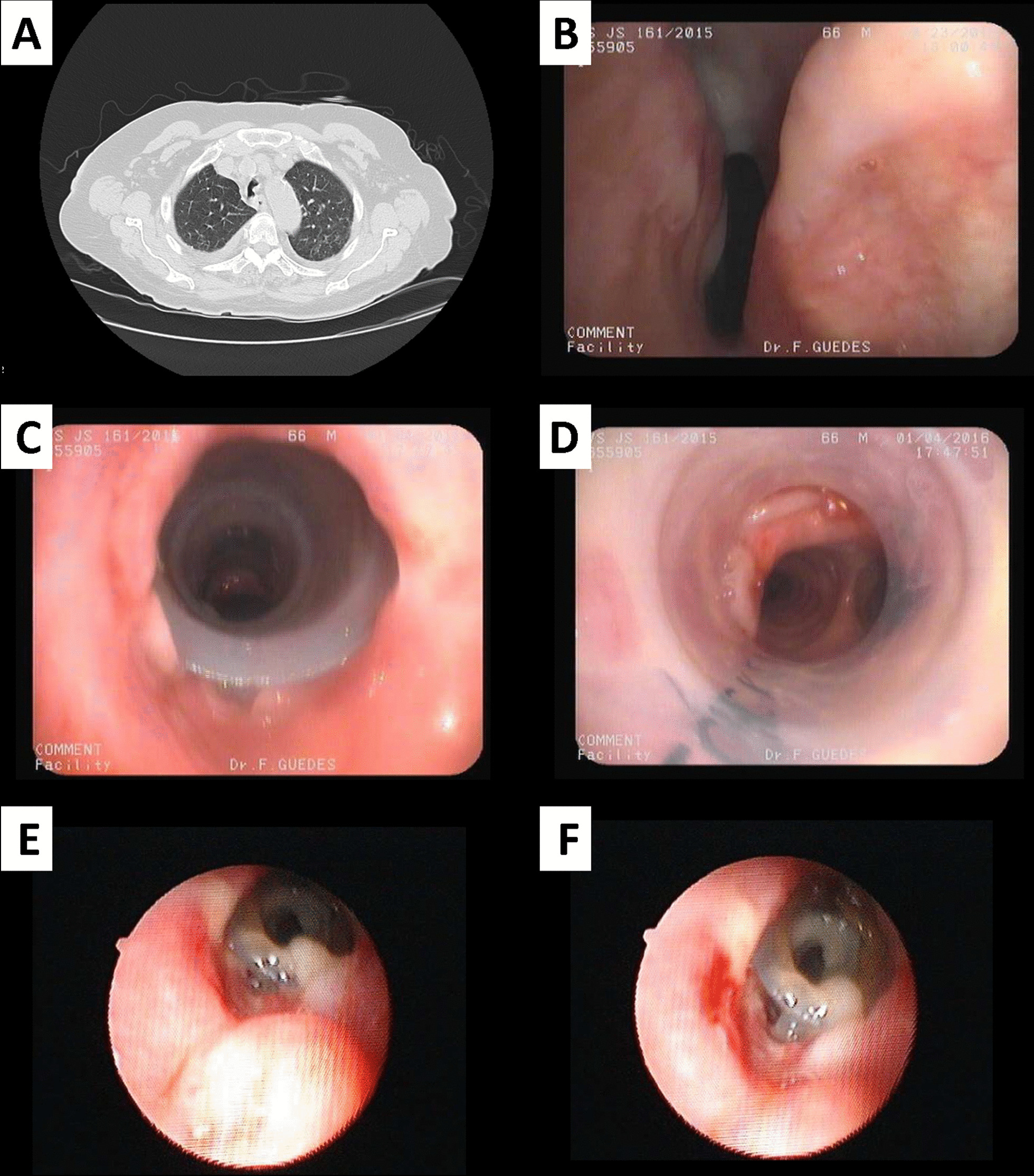
**A** CT scan shows a narrowing in the mid trachea; **B** Endoscopic vision of subglottic; **C** endoscopic view of trachea after placement of stent, showing the patency of airway; **D** Distal view of stent; **E**, **F** Stent migration and partially obstructed by mucus. **E**, **F**, Courtesy of Dr. José Almeida – Centro Hospitalar de Vila Nova de Gaia, Portugal

**Fig. 3:**
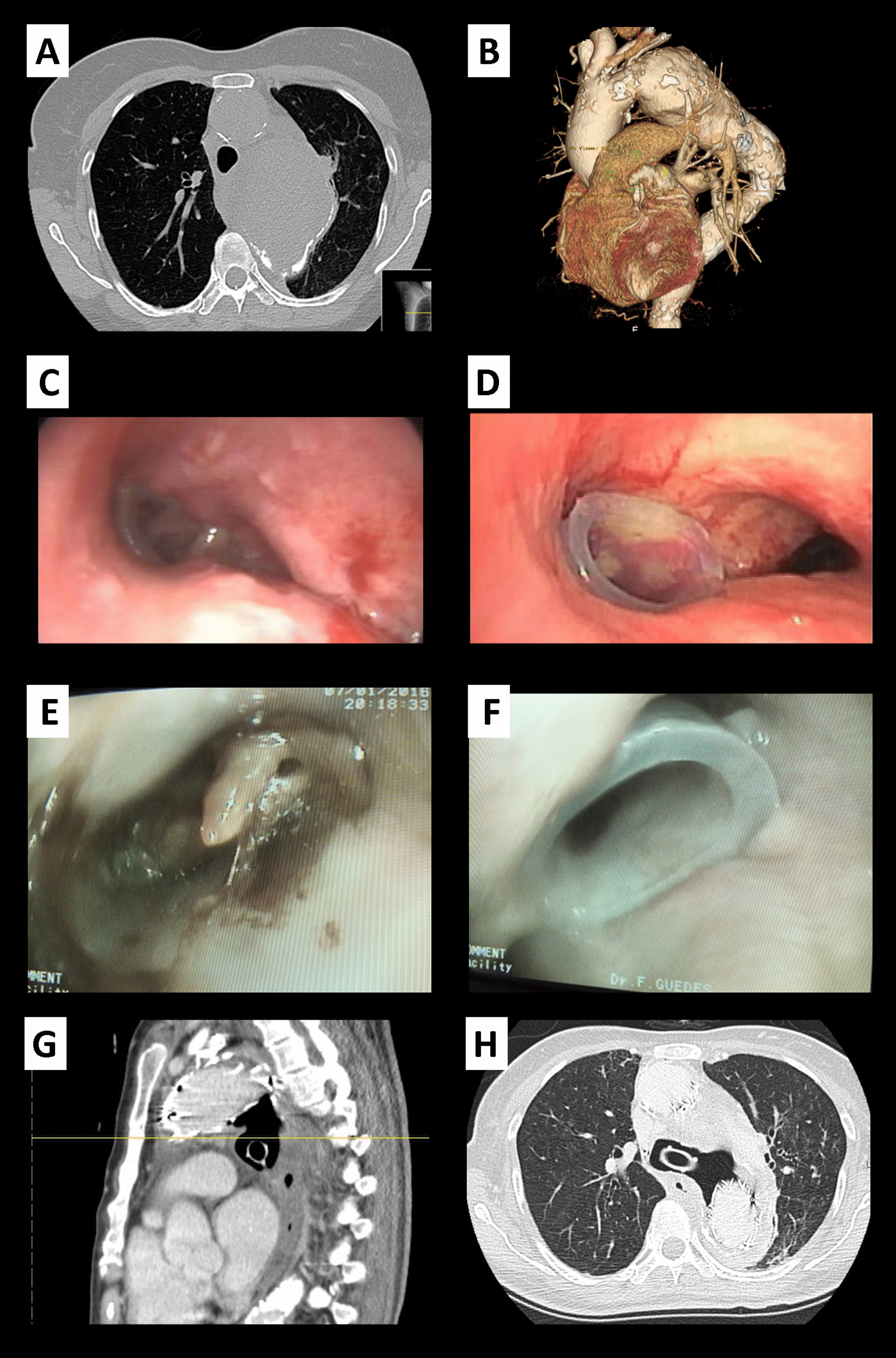
Sequence of happenings observed in thoracic CT scan and in bronchoscopy: **A** thoracic aorta aneurism with tracheal contact; **B** 3-D reconstruction of thoracic aorta aneurism; **C** carina with signs of extrinsic compression; **D** stent in left main bronchus; **E** total occlusion of stent witch mucus; **F** patency of stent post laborious cleaning; **G**, **F** evidence of fistulization from LMB to mediastinum, contacting directly to aneurismatic sac

**Fig. 4:**
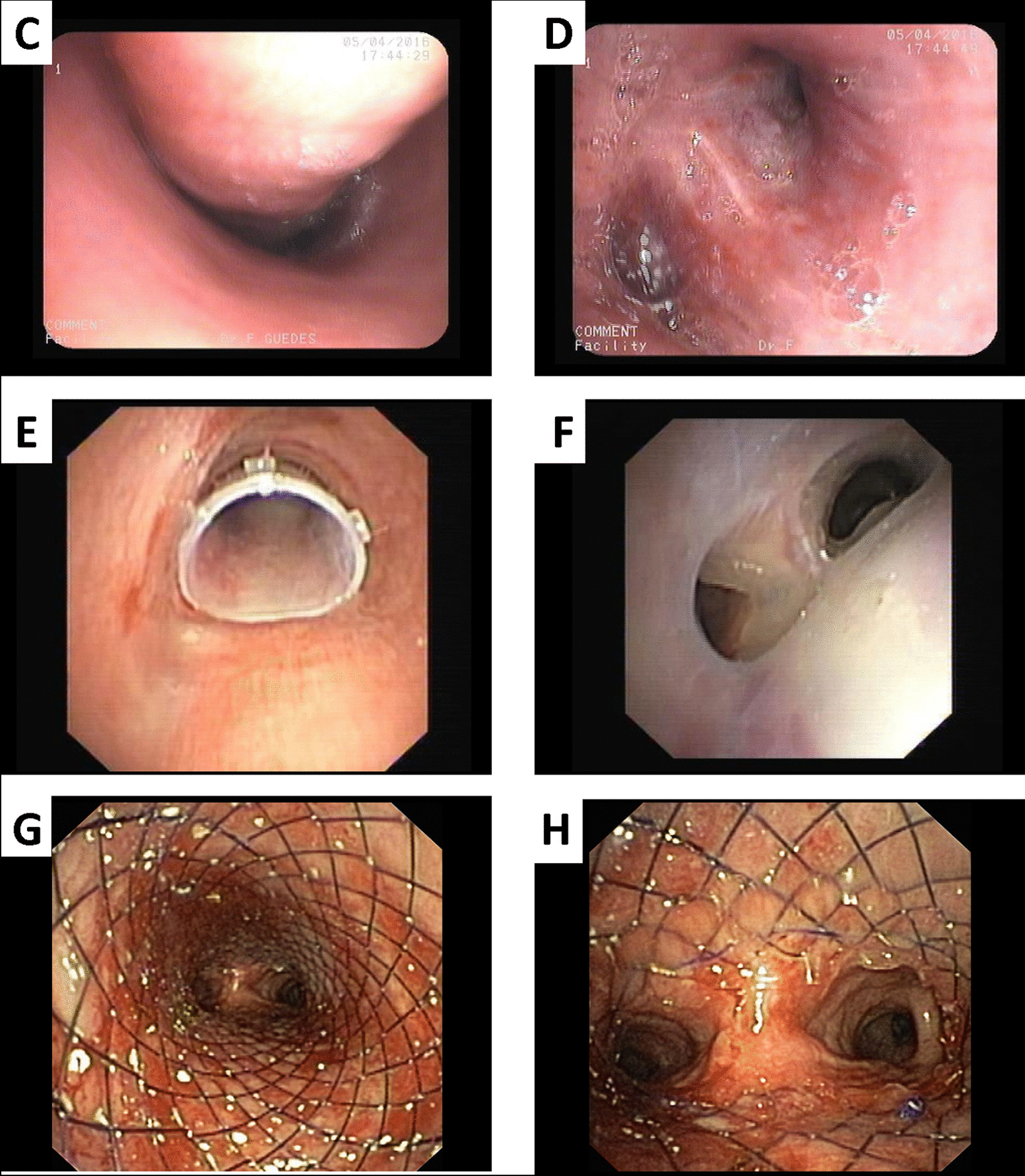
**A**, **B** Thoracic CT scan sowing voluminous adenopatic conglomerate in mediastinum com compressing trachea and both main bronchi; **C** Endoscopic view of extrinsic compression of trachea; and **D** Left main bronchi; **E** Proximal view stent in trachea; **F** Inside Y stent showing left and right portion of stent

## Discussion on CAO management and the role of tracheal stents

CAO cases must have a multidisciplinary team discussion to cover all possible treatments and to select the best therapeutic options. Therapy must be addressed according to the pathologic findings and to the patient´s health status. Definitive management may include both medical and surgical interventions. Possibilities embraces observation, drug therapy, radiation, tracheostomy, endoscopic treatment, and surgical management. Endoscopic treatment includes dilatation, laser resection and airway stenting [62]. As described before, several classification systems can be used to predict the success of the intervention, although further studies are needed to confirm its usefulness. In non-symptomatic or minimal symptomatic patients with stable and non-progressive stenosis, close observation is considered an adequate approach [63]. In the decision of management, according to Galluccui et al., the most important differentiation to be made is between simple and complex stenosis as it determines the success or failure of the endoscopic intervention [64]. Complex tracheal stenosis is associated with extensive scaring (> 10 mm) and varying degrees of cartilage involvement, circumferential contraction scaring, or tracheal stenosis associated to *malacia* or inflammation. Tracheal sleeve resection is considered to be the first-option and definitive treatment in complex tracheal stenosis [65]. Nevertheless, bronchoscopic management in selected patients is reported to have a high success rate [66, 67]. Thus, a short segment, membranous, benign, concentric and not involving cartilage stenosis can be consider for endoscopic treatment as first option [30]. It is also an option for patients with surgical ineligibility due to the stenosis characteristics, infection, or poor medical status. Complex stenosis may require stent placement. Endoscopic intervention can also work as a bridge for surgical treatment [64]. In the first clinical case, the patient had a complex stenosis. Although surgery could not be performed because of comorbidities, the decision based on the difficult mechanical ventilation, easily reaching high plateau pressures with low tidal volumes, was taken with the purpose of being only temporary. Prior to this scenario, the patient manifested mild symptoms for 2 years, and at the time, the option was the maintenance under close observation but without intervention. Interventional bronchoscopy options include the use of both rigid and flexible bronchoscopy. Both have advantages in different scenarios and should be seen as complimentary procedures [62, 68]. Guidelines do not favour rigid over flexible bronchoscopy, although the procedure with rigid bronchoscopy is considered safer. Through the flexible bronchoscope thermal and cryotherapy, balloon dilatations, micro-debridement and stenting can be performed. Several thermal techniques can be used [61]: (a) electrocautery, one of the most available and cheap, achieves an immediate outcome but requires a skilled user, also requires contact and FiO_2_ should be decreased_;_ (b) argon-plasma coagulation is a non-contact technique capable to burn tissue and control bleeding. It has a penetration depth of 2-3 mm, which makes it a safe but longstanding technique; (c) LASER has supplanted other thermal techniques because of its minimum energy delivery and cut precision. CO_2_ laser is more precise but has a limited coagulation effect compared with Nd:Yag laser[69]. In contrast to heat therapies, contact cryotherapy has been also described as useful in CAO. It is exclusively used for endobronchial lesions and may require repeated procedures. Rigid bronchoscope debulking of lesions and scar tissue is also simple, effective and accomplished by suction under direct telescopic guidance [62, 68].

Several drugs used to prevent the complications associated to endoluminal wound healing have been assessed in the last decades and are still under investigation. The most used as co-adjuvant are corticosteroids [70] and mitomycin-c. A literature review in 2010 concluded that mitomycin-c may postpone but not prevent the recurrence of stenosis [71].

For patients with primary lung cancer which develop CAO, besides all interventional bronchoscopy techniques mention above, in some cases, additional stenting is required [68]. Airway stents are tube-shaped devices that are inserted into the airway to maintain patency. Stent placement is indicated in both intraluminal and extraluminal significant CAO and can be used in benign and malignant disease. Placing an airway stent is a difficult decision, and the operator must consider the benefit of its application, by preventing the airway re-occlusion, and also its disadvantages, mostly regarded with long-term associated complications [72, 73]. There are two main types of airway stents concerning the material they are made of: tubular stents made of silicone and SEMS. Metallic stents come with or without a silastic or polyurethane covering, which are used to minimize tumour overgrowth and tissue granulation [74]. The development of granulation tissue is one of the common occurrences in uncovered or partially covered stents, and its coverage was developed to limit this disadvantage. Indeed, in covered stents, granulation tissue tends to only be located at the extremities and its progression into the stent is rare [75]. Both types of stents can be placed through rigid bronchoscopy, although SEMS could also be placed with flexible bronchoscopy. As a foreign body, there are complications associated to endotracheal stents (Table 3).

**Table 3:** Complications associated with silicon and metallic stents

Potential complications of endotracheal stents	Silicon stent	Uncovered metallic stent	Covered metallic stent
Stent migration	++	–	–
Disruption of mucociliary clearance	++	–	+
Mucous plugging	++	–	+
Recurrent infection	+	+	+
Cough	+	+	+
Granulation tissue formation	+	++	+
Stent Fracture	–	++	+
Fistula formation	+	++	+

++ Very usual, + Usual, – Unusual

Metallic stents, especially non-covered, should be considered permanent because of extensive granulation tissue and epithelialization. The longer the stent remains, the more difficult is its removal. Silicone stents are more easily replaced or removed [76]. Based on 2005 Food and Drug Administration warning, metallic stents are not recommended in benign airway strictures because of the reported extensive granulation tissue and stent fracture. Considering these recommendations, they should not be placed in patients where removal is considered in the future [32, 77]. In the 3 cases presented, disrupt of mucociliary clearance and mucus plugging were detected as clinical impairments. Infection aroused in 2 cases and migration in the first clinical case. Fistulation is a serious problem, and happens because of friction between stent and mucosa [78]. In TBM, stent insertion should be only applied in patients with symptomatic and severe obstruction (> 60%) and non-responsive to positive pressure ventilation. QOL and functional status are improved in short term in 70% and dyspnea in 90% of patients after silicone stenting [79, 80]. For patients with severe, diffuse and symptomatic TBM who had repeated stent migration, external stent fixation, tracheostomy or Montgomery T-tubes are a good option to improve symptoms or as a bridge to a more definitive treatment [79]. As the ideal stent is still not available, physicians should consider the full assortment of silicone and SEMS. The main advantages of silicon stents rely on their easy removal and repositioning, less granulation tissue formation and absence of tumour ingrowth. On the other hand, rigid bronchoscopy is needed for insertion, and they are more suitable to migrate. SEMS are easy to insert, as it can be performed by flexible bronchoscopy. Following, they are more stable and more effective in extreme extrinsic compression. The main disadvantages are the impossibility of repositioning or removal and tumour ingrowth [50], especially in the case of uncovered metallic stents. The ideal stent should be simple to insert, easily removed, capable to resist compression and to allow clearance of secretions, as well as easy to nail and avoid migration. New custom-made and bioabsorbable airway stents made of different biomaterials are under investigation and have been placed in humans. Polydioxanone is the one that has been most often used and for the longest period, with report of a patient successfully treated and free of intervention for 44 months [81, 82]. Other biomaterials are under investigation, as the polylactic acid, polyglycolic acid, polycaprolactone and polyamide[83, 84]

## Outcomes

In malignant diseases, there is no controlled trial evidence regarding tracheal or bronchial intervention due to ethical concerns. However, it is consistently demonstrated in the literature that interventional bronchoscopy procedures with stenting improves symptoms of dyspnea, QOL and lung function [2, 85, 86]. Long term favourable outcomes are achieved with a combination of laser and gentle dilatation in benign tracheal stenosis [32, 64, 87]. Although the use of stents in benign clinical conditions is not consensual, a considerable number of supportive literature describes promising results [88]. According to Monnier et al., using the Myer-Cotton grading system, the success of interventions with CO_2_ laser is 92% in grade I, 46% in grade II and 13% in grade III [17]. Lim SY and colleagues, concerning PIS, demonstrated that the time relapsed between stenosis and first intervention is important, with positive outcomes of 90% for those with intervention within 6 months and 61% for those with longer intervention period [89]. According to Galluccio et al. the use of silicone stents as part of endoscopic intervention, achieved airway patency of 96% in simple stenosis and 69% in complex stenosis [64]. When stents are used in TBM, the complication rates are higher when the disease occurs with loss of cartilaginous support [64].

## The role of tracheal tissue engineering in CAO

Airway tissue engineering is a field of Regenerative Medicine with the goal of developing biological substitutes that can restore, maintain, or improve tissue functions. When stenting is not possible, there are other options (Table 4) with pros and cons that range from autografting and allografting to the use of prosthetic materials. Prosthetic options have some limitations such as development of inflammation and incomplete host tissue integration. In the case of grafts, autografts are good options due to the absence of rejection and inflammation after application, however the availability of donor tissue is a limitation. Furthermore, allografts must be associated with immunosuppressive drugs. To overcome these constraints, alternatives associated with tissue engineering have been developed in recent years. In these approaches, therapies are based on the use of three essential elements: cells, growth factors and scaffolds in distinct combined approaches [90]. Different cell populations have already been extensively applied in this field. Tissue-engineered tracheal bioprosthesis coated with chondrocytes and polymers were used and implanted in vivo, and Mesenchymal stem cells (MSCs)-derived chondrocytes have also been considered as alternative cell sources, particularly in promoting tracheal cartilaginous tissue regeneration [91]. These cells allow biomechanically stable seeded scaffolds, but chondrocytes become infeasible after implantation. Epithelial cells were explored as an alternative, and scaffolds seeded with these cells demonstrated increased viability and promoted better tracheal regeneration in vivo [92]. To maximize the advantages of each cell type, a combination of epithelial cells cocultured with articular chondrocytes was tested, and good in vitro performance, as well as positive tissue regeneration in vivo*,* were observed [93]. The co-culture of bone marrow derived MSCs with fibroblasts and endothelial cells of the cartilage and umbilical vein, their aggregation in spheroids and subsequent 3D printing in an architecture similar to native tracheal tissues allowed the development of layers of epithelial tissue with good viability and vascularization [94]. The culture of Induced pluripotent stem cells in an air—liquid interface system allowed their differentiation into ciliated epithelial cells and non-ciliated club cells, making them fair candidates for application in tracheal regeneration [95]. Tissue engineered airway grafts seeded with isolated cells from umbilical cord blood, amniotic fluid or adipose tissue revealed to not be subjected to immune rejection and can provide mechanical characteristics compatible with tracheal physiology. Finally, the use of cells from fetal tissues allowed the synthesis of a cartilage rich in glycosaminoglycans, β-elastin and smooth muscle cells, with epithelial growth, smooth muscle having cilia movement and no ectopic growth or tumour development [90, 96]. The scaffolds to be used to promote regeneration of tracheal tissue must have some specific characteristics, namely: design that mimics the anatomical shape of the trachea or defects to be filled; mechanical strength and flexibility identical to native tracheal tissue to prevent collapse; porosity that allows good vascularization and cell proliferations. In addition, the scaffold must be biocompatible, biodegradable, non-toxic and non-immunogenic, to ensure a good cytocompatibility and to not trigger organic reactions and rejections [97]. To guarantee these characteristics, the scaffold manufacturing methods as well as their material are important decisions to consider. Various natural and synthetic materials have already been used, either alone or in combination, including silk, gelatin, polyglycolic acid, collagen, polycaprolactone and decellularized matrix [90]. Growth factors influence cell proliferation and differentiation by modulating the pro-regenerative environment at the site of administration, also ensuring cell growth and nutrition. The list of growth factors already used in tracheal tissue engineering include insulin-like growth factor, transforming growth factor, granulocyte colony-stimulating factor, vascular epithelial growth factor, basic fibroblast growth factor, epithelial growth factor and platelet-derived growth factor [90]. These growth factors can be administered both exogenously and loaded on the scaffold, but while the first approach is more advantageous in the case of minor injuries, endogenous administration is most appropriate in significant tracheal damages [98]. The best combination of cells, scaffolds and growth factors has not yet been defined, but tissue engineering has the potential to allow the development of alternative methods for effective tracheal reconstruction. The goal of these technologies is the development of a fully autologous engineered trachea that allows to overcome the limitations of the current synthetic and grafts options.

**Table 4:** Advantages and disadvantages of Tissue Engineering techniques currently being explored for tracheal reconstruction

	Advantages	Disadvantages
Prosthetic material	Physical and functional replacement of the trachea	Risk of inflammation Incomplete host tissue integration
Autografts	Absence of rejection Absence of inflammation	Donor tissue availability
Allografts	Availability of donor tissue	Risk of intense inflammation Risk of organic rejection Dependence on immunosuppressive drugs
Cell-based therapies	Can be used in combination with prostheses, scaffolds, and growth factors Promotion of in vivo tracheal regeneration Specific cell differentiation	Inconsistent results The ideal cell type to be used has not yet been identified Risks of organic rejection Risks of genetic instability Risks of tumorigenic differentiation Difficulties in isolation and cell expansion
Scaffolds	Availability of materials with synthetic and natural origin Can be used in combination with cells to maximize their benefits	High manufacturing requirements Requirement of precise physical characteristics to guarantee cytocompatibility, biocompatibility, biodegradability, and absence of local and systemic toxicity
Growth factors	Can be used in combination with cells to maximize their benefits Influence cell proliferation and differentiation Ensure cellular growth and nutrition Modulation of the pro-regenerative environment at the site of injury and site of administration	Risk of adverse reactions Challenging manufacturing techniques Compromised product stability Inconsistent results

## Conclusion

CAO represents an important clinical impairment caused by benign or malignant diseases. Surgical management remains the preferred approach but there are situations in which, whether due to patient limitation or the nature of the pathology, intervention bronchoscopy, with its accessory techniques such as laser, cryotherapy, mechanical dilation, or debridement, play a preponderant role. When it comes to the choice of a stent, several considerations must be considered. Silicone stents are cheaper, easily retrievable and can be repositioned as much times as necessary. On the other hand, they need for rigid bronchoscopy to be handled, are more suitable to migrate and interfere with mucociliary clearance. Metallic stents can be placed by flexible bronchoscopy, are usually more adaptable to an irregular surface and allow reepithelization. The same way, the tumour ingrowth can happen which make them almost impossible to be securely removed. Although an exciting new generation of personalized airway stents along with stem cell therapy is expected, this reality is far away from being achieved. A recent evolutional step was made when manufacturers offered modifications of their products according to patient´s needs, with rapid prototyping 3D printing techniques, allowing stents to be tailored to the individual´s airway in days or even hours [99]. If on one hand 3D printing technology could become accessible for physicians, on the other hand there are no certified manufacturers nor a direct access between parts. Maybe a creation of a software easy-to-use between hospitals and manufacturers could provide that answer but there are still ethical and legal issues to overcome [100].

## Data Availability

The data that support the findings of this study are available from the corresponding author on request.

## Abbreviations

c-ANCA: Cytoplasmic autoantibodies
CAO: Central airway obstruction
COPD: Chronic obstructive pulmonary disease
CT: Computer tomography
LMB: Main left bronchus
LTS: Laryngo-tracheal stenosis
MRI: Magnetic resonance imaging
MSCs: Mesenchymal stem cells
PITS: Post-intubation tracheal stenosis
PSST: Post-tracheotomy tracheal stenosis
QOL: Quality of life
RP: Relapsing polychondritis
SEMS: Self-expandable metallic stents
TBM: Tracheobronchomalacia
TO: Tracheobronchopatia osteochondroplastica
